# Recognition of masked and unmasked facial expressions in males and females and relations with mental wellness

**DOI:** 10.3389/fpsyg.2023.1217736

**Published:** 2023-10-30

**Authors:** Marie Huc, Katie Bush, Gali Atias, Lindsay Berrigan, Sylvia Cox, Natalia Jaworska

**Affiliations:** ^1^University of Ottawa Institute of Mental Health Research, Ottawa, ON, Canada; ^2^Department of Neuroscience, Carleton University, Ottawa, ON, Canada; ^3^Department of Psychology, St Francis Xavier University, Antigonish, NS, Canada; ^4^Department of Psychiatry, Dalhousie University, Halifax, NS, Canada; ^5^Department of Psychiatry, McGill University, Montreal, QC, Canada; ^6^Department of Psychology, Dawson College, Montreal, QC, Canada; ^7^Department of Cellular & Molecular Medicine, University of Ottawa, Ottawa, ON, Canada

**Keywords:** emotion, face mask, sex differences, mental health, loneliness, anxiety/stress, depression

## Abstract

**Background:**

While the effects of mask wearing/facial occlusion are known to impair facial expression recognition, little is known about the role of mental wellness on facial expression recognition, as well as the influence of sex on misattribution errors (i.e., confusions between emotions). In this large study, we aimed to address the relation between facial expression recognition and loneliness, perceived stress, anxiety, and depression symptoms in male and female adults.

**Methods:**

We assessed the influence of mask-wearing on facial expression recognition [i.e., accuracy and response time (RT)] via an online study in *N* = 469 adult males and females across Canada.

**Results:**

Expectedly, recognition was impaired under masked conditions (i.e., lower accuracy, longer RTs, more misattribution errors). Females were faster and more accurate than males, with less misattribution errors. A novel finding was that people with higher perceived stress were less accurate at identifying masked fearful faces. Perceived stress influenced the relation between sex and RT to masked happy faces; males with high stress scores were slower to recognize masked happy faces, the opposite was true for females. Finally, this study was among the first to show that higher loneliness predicted shorter RT to unmasked faces.

**Impact:**

Our results show that facial expression recognition is impaired by mask-wearing, and that sex and mental health features are important predictors of performance. Such insight could be detrimental in certain sectors of the population (e.g., health care or education), and inform policies being adopted in future pandemics.

## 1. Introduction

Facial expressions play a central role in non-verbal communication thereby facilitating social interactions. Impairments in correctly recognizing facial expressions can lead to social interaction challenges (Bistricky et al., 2011). Previous work indicates that areas around the eyes and mouth provide important cues for the successful recognition of specific emotions. Upper facial features seem particularly important in recognizing sad, fearful and angry faces while lower features play a more pivotal role in happiness, surprise and disgust recognition (Wegrzyn et al., 2017).

Expectedly, facial feature obstruction impedes expression recognition (Roberson et al., 2012). As such, mask-wearing mandates issued by many countries to minimise the spread of the COVID-19 virus for several years created challenges to facial expression recognition and, by extension, to non-verbal communication. While public mask-wearing was common in certain nations pre-COVID-19 for limiting the spread of communicable diseases, regular public mask-wearing in most Western nations was rare (Zhang et al., 2022). As such, large swaths of the global population were, rather suddenly, impeded in their ability to communicate through facial expressions. Recent studies have corroborated impaired facial expression recognition with facial coverings (e.g., Carbon, 2020; Gori et al., 2021; McCrackin et al., 2022). Further, misattribution errors (i.e., confusing one emotion with another) are higher under masked vs. unmasked conditions (Carbon, 2020; Tsantani et al., 2022). The most common misattribution errors include the confusion of fearful with surprised faces (Roy-Charland et al., 2014) and angry with disgusted faces (Wegrzyn et al., 2017). Interestingly, some work indicates that masks result in more emotional expressions being confused with neutral ones (Kim et al., 2022).

Several studies have reported that, on average, females vs. males are faster and more accurate in identifying facial expressions (Connolly et al., 2019). Recent research also found that females were more accurate in recognizing facial expressions of emotions even with mask occlusion, particularly surprised and sad faces (Grundmann et al., 2021; Proverbio and Cerri, 2022), though exceptions exist (Kim et al., 2022). In a meta-analysis, Thompson and Voyer (2014), observed larger sex differences among younger cohorts (i.e., teenagers/young adults) compared to older individuals, with the largest advantage observed for young adult females. Other studies suggest that older adults are less accurate in recognising emotions than younger adults (Abbruzzese et al., 2019), though this age effect might be more pronounced for negative emotions (Calder et al., 2003). Thus, age (and/or an age-by-sex interaction, e.g., Abbruzzese et al., 2019) is an important variable to consider when assessing facial expression recognition.

During the COVID-19 pandemic, many countries implemented social restriction measures (e.g., distancing) which likely contributed to negatively impacting wellbeing in the general population (Salari et al., 2020), particularly among young adult females (McQuaid et al., 2021; Prowse et al., 2021). Specifically, a rise in loneliness, distress, anxiety and depression symptoms have been reported (Salari et al., 2020). However, there is limited research assessing if this deterioration in wellbeing is associated with facial expression recognition. Previous studies noted that more (vs. less) lonely individuals were better at recognizing emotions (Vanhalst et al., 2017), particularly sadness (Cheeta et al., 2021), though others reported no relation between loneliness and emotion recognition (Lodder et al., 2016).

The link between perceived stress and emotional processing is unclear, though stressors can influence facial expression recognition. For instance, acute psychosocial stressors have been linked to increased facial emotion recognition performance (Domes and Zimmer, 2019), though this might depend on the emotion displayed (Daudelin-Peltier et al., 2017). Additionally, individuals with anxiety disorders exhibit a moderate impairment in facial expression recognition (Demenescu et al., 2010), although this may be specific to fearful faces (Doty et al., 2013). As such, affect, and by extension, dysregulated mood, appears to influence facial emotion processing and recognition (Lawrie et al., 2019). The mood-congruency hypothesis posits that if a person is in a sad/negative mood state, for instance, the perception of sad faces might be facilitated (Schmid and Schmid Mast, 2010); this theory has been used to explain why depressed individuals tend to display enhanced recognition of sad facial expressions and/or attribute negative affect to neutral expressions compared with non-depressed individuals (Suslow et al., 2020; Monferrer et al., 2023).

To date, little is known about the effects of loneliness, stress, anxiety, and depression symptoms on facial expression recognition with and without masks, and the influence of sex on these relations. Given that mask-wearing may become more normalized in the future (e.g., as part of public safety measures during pandemic outbreaks), understanding the relation between facial expression recognition and wellbeing is topical as it might inform future public policy measures and/or training programs.

In this online study (*N* > 450 adults), we assessed the effect of sex on facial emotion recognition with and without masks, while controlling for the potential confounder of age. We also examined the effect of mask-wearing and sex on misattribution errors. Novel contributions included assessments of the effects of loneliness, perceived stress, anxiety, and depression symptoms on masked and unmasked facial expression recognition. We aimed to test the following:

1) We aimed to replicate the impact of masks on facial recognition. We predicted better performance and less misattribution errors for unmasked faces.
2) We assessed putative differences between males and females in facial emotion recognition. We expected an advantage for females (i.e., better performance and less misattribution errors), regardless of unmasked/masked condition.
3) Most novel, we examined the impact of mental health symptoms on facial emotion recognition of masked and unmasked faces, and whether these symptoms impacted males and females’ performances differently. We anticipated that better overall emotional recognition would be associated with lower stress, anxiety, and depression symptoms (relation with loneliness was unclear), although recognition of mood-congruent expressions may be improved (e.g., better recognition of fear with higher levels of anxiety symptoms). Finally, we had no directional hypotheses about whether mask-wearing or sex would alter these relations as this has not been previously examined, to our knowledge.

## 2. Methods

### 2.1. Transparency and openness

We report how we determined our sample size, all data exclusions, manipulations, and measures in the study. The study reported in this article was not preregistered. Requests for the data can be sent to the corresponding author.

### 2.2. Procedure

The web address to the study, delivered using the online platform Gorilla®(Anwyl-Irvine et al., 2020), was distributed to students/staff at universities and hospitals affiliated with the researchers via email, and various social groups/networks within the researchers’ communities (e.g., volunteer organizations). The study was also posted on social media accounts (e.g., special student groups). Data was collected between February 2021 and 2022. Only respondents living in Canada were included. In line with the legal age of consent to participate in research in Canada, respondents <18 yr. were excluded. Individuals suffering from any self-reported conditions that could influence facial expression processing were also excluded (e.g., stroke). Following successful completion of the study, participants could redeem a 10CND electronic gift card. This research received approval by the Research Ethics Boards of the ROMHC, St. Francis Xavier University and Dawson Research Ethics Board (REB#s 2020024; 25316; JAWON2021181).

### 2.3. Questionnaires

The online study commenced by confirming Canadian residency; socioeconomic and demographic information was then gathered. Participants were asked about current medical conditions and psychiatric/psychological problems. The 9-item Patient Health Questionnaire (*PHQ-9*, e.g., “*Little interest or pleasure in doing things*” with answers on an ordinal scale from “1. Not at all” to “4. Nearly everyday”) (Kroenke et al., 2001) was used to assess depressive symptoms while the 7-item Generalized Anxiety Disorder Scale (*GAD-7,* e.g., “*Feeling nervous, anxious or on edge*,” with answers from “1. Not at all” to “4. Nearly everyday”) (Spitzer et al., 2006) was used to assess anxiety symptoms, in both cases over the last 2 weeks. Loneliness over the last month was measured with the 8-item UCLA Loneliness Scale (*ULS,* Likert scale: e.g., “*I lack companionship*” with answers from “1. I often feel this way” to “4. I never feel this way”) (Hays and DiMatteo, 1987) and perceived stress over the last month was measured using the 10-item Perceived Stress Scale (*PSS,* e.g., “*In the last month, how often have you felt nervous and ‘stressed’?*” with ordinal scale answer from “1. Never” to “5. Very Often”) (Cohen et al., 1983). Questionnaires took ~15 min to complete.

### 2.4. Emotional processing tasks

**Figure 1:**
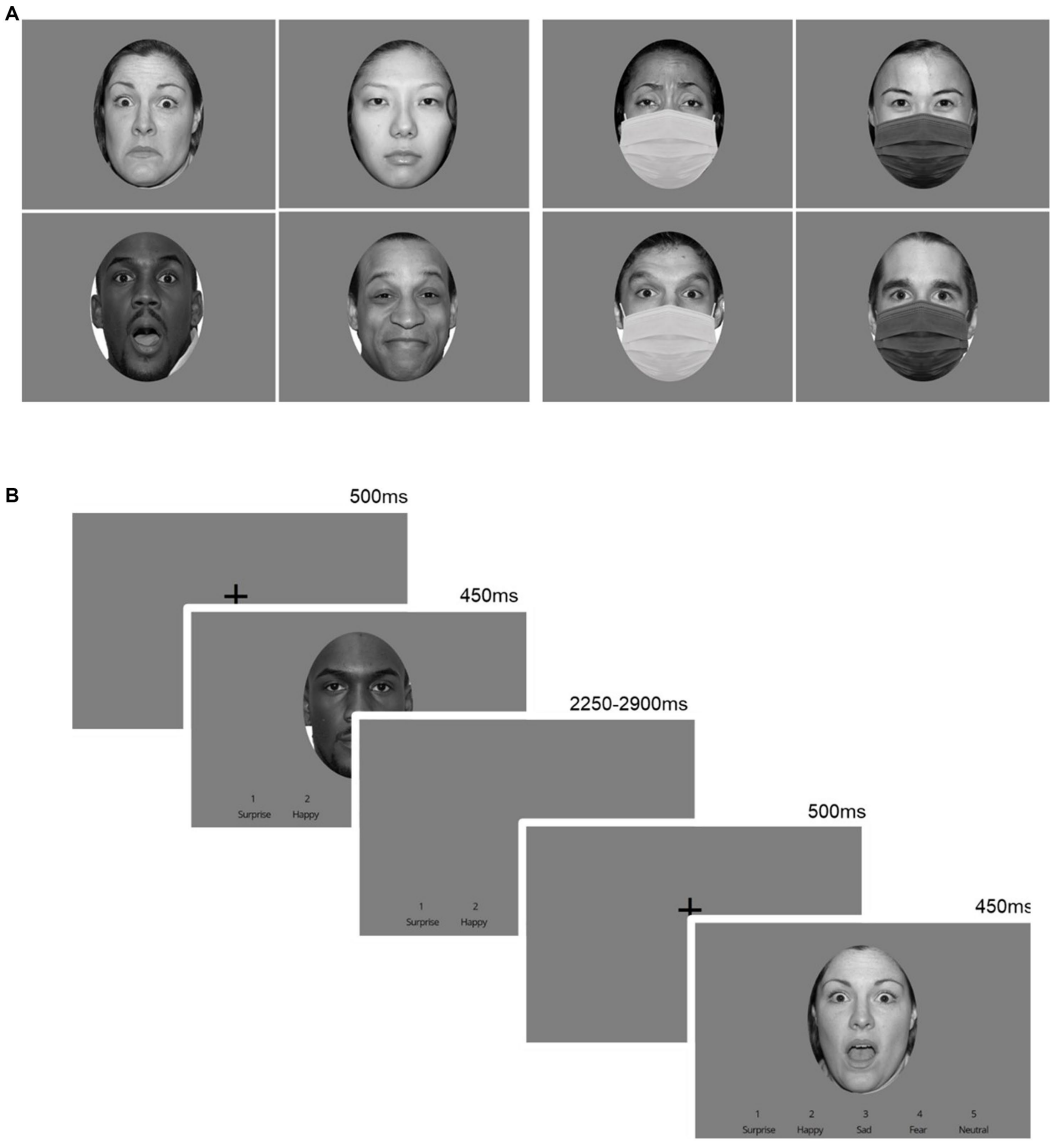
Emotional processing tasks. **(A)** Two versions of the tasks were created with masked or unmasked facial expression conditions using pictures of actors selected from the NimStim dataset, adapted from “The NimStim set of facial expressions: Judgments from untrained research participants” by Tottenham et al. (2009), Copyright 2008 by Elsevier Ireland Ltd. Reproduced with permission of Elsevier. **(B)** A trial consisted of a fixation cross (500 ms), followed by the stimuli (450 ms), and a response window (2,250–2,900 ms, mean: 2,500 ms).

Two versions of the task, one with and one without masks occluding the lower half of the face (white/black masks overlaying black-and-white photos of actors; Figure 1A) were presented one after the other following questionnaire completion; the order of the tasks was randomized. Comparable to others’ (Langenecker et al., 2005; Jenkins et al., 2018; Saylik et al., 2018), participants were instructed to respond as quickly and accurately as possible when identifying a facial expression by pressing a corresponding keyboard key; key order was randomised across participants (e.g., 1 = surprise, 2 = happy, 3 = sad, 4 = fear, 5 = neutral). Each condition version (masked/unmasked) consisted of 120 trials over 3 blocks (block randomization design, with 40 trials randomized/block). Each block consisted of faces expressing the five emotions selected from the NimStim dataset (Tottenham et al., 2009; Supplementary material). Images of 4 male and 4 female actors (White, Asian, Latino and Black) were used to express all 5 emotions (40 images total, each repeated 3 times). All images were grey-scaled, identical in size, matched for luminance, with hair and neck circularly cropped out. No identical images were presented back-to-back. Each block began with instructions (Supplementary Figure S1), followed by a practice block (10 actors, different from those used in tasks). For each trial, a fixation cross was first presented (500 ms), followed by a face (450 ms). Subsequently, a response window (blank/grey screen) followed (2,250–2,900 ms, mean: 2,500 ms). Participants could respond during the presentation of the face or response window (Figure 1B). The number of correct, incorrect, and missed responses as well as correct response times (RT) were recorded. Accuracy was calculated as the addition of all correct answers.

### 2.5. Quality controls and statistical analyses

Data analyses were conducted using SPSS (Version 27.0, IBM). To ensure that participants were not indiscriminately responding, quality controls were performed using similar questionnaire items. Namely, items 1 and 2 on the *PHQ-9*, items 2 and 3 on the *GAD-7*, and items 1 and 5, 4 and 5, and 2 and 8 on the *UCL* (i.e., 5 separate quality controls) were compared for consistency. Difference scores of ±3 were flagged; if there were > 2 “flags,” participants were removed (no participants removed as a result of these checks). Bots were identified using two quality control questions between questionnaires (e.g., “Write the name of an animal in the following box”), a visual search task after the tasks (i.e., “You will have to find the cat and click on it”) or during the tasks with repeatedly entered keys (e.g., >100 different keys entered per stimuli). A total of 135 bots were removed. Data from both tasks were removed for 15 participants (*N* = 10F; *N* = 5 M) because of missing data. The final sample was *N* = 469 (120 M/349F).

Normality and sphericity were assessed using Shaprio-Wilk and Mauchly tests, respectively. Non-parametric tests were carried out if normality was violated. If sphericity was violated, a Greenhouse–Geisser or Huynh-Feldt correction was applied, as appropriate (Howell, 2002; Field, 2013). Extreme outliers (ZScore> ± 3.29) were identified and replaced by the nearest value that is not considered extreme per variable. To create a combined “performance score,” the following formula was used: $z_{ACC_{i,j}}$ +[(−1)*( $z_{RT_{i,j}}$ )] (Moore et al., 2015; Vandierendonck, 2021). A higher score corresponds to better performance, and takes into account the speed-accuracy trade-off (Liesefeld and Janczyk, 2019). Performance scores are presented in Supplementary material as they were comparable to accuracy.

Age, education (yrs), sex, ethnicity and mental health status and wellness scores among males and females were compared with Chi-Square or Mann-U Whitney tests (Table 1). Repeated measures analyses of covariance (rmANCOVAs) were used, with condition (masked/unmasked) and emotion (fear/happy/neutral/sadness/surprise) as within-subject factors, while respondent’s sex (males/females; instead of respondent’s gender as too few [*N* < 10] did not identify with their biological sex although they were still included in this study) was the between-subject factor for RT to correct responses, accuracy and performance scores. Initial analyses showed no effect of participant’s ethnicity or ethnicity of the stimuli/actors on emotion facial recognition (data not shown). Therefore, ethnicity were not included in the analyses. Initial analyses revealed a main effect of age on outcomes (data not shown); thus, age was used as a covariate in rmANCOVAs analyses. Bonferroni-corrected pairwise comparisons were used to follow-up significant (*p* < 0.05) main effects/interactions. For 2/3-way interactions, only results that are not represented in the main effects are reported. Partial eta square ( $\eta_{p}^{2}$ ) was included as a measure of effect size.

**Table 1:** Participant characteristics (means and standard deviation presented).

	Males (*n* = 120)	Females (*n* = 349)	Mann–Whitney or *χ*^2^
**Age**	33.02 (14.44)	32.65 (13.38)	*z* = −0.20, *p* = 0.84
**Years of education**	15.77 (2.50)	15.96 (2.30)	*z* = −1.33, *p* = 0.18
**Gender**			
Cisgender	98.3% (*n* = 118)	98.3% (*n* = 343)	*χ*^2^(1) = 0.001, *p* = 0.97
Other	1.6% (*n* = 2)	1.7% (*n* = 6)	
**UCLA Loneliness Scale** (ULS, 8-item)	18.96 (4.96)	19.63 (4.67)	*z* = −1.05, *p* = 0.29
**Perceived Stress Scale** (PSS, 10-item)	17.18 (7.28)	20.04 (6.77)	*z* = −3.64, *p* < 0.001***
**Patient Health Questionnaire** (PHQ, 9-item)	7.37 (5.69)	8.07 (5.61)	*z* = −1.44, *p* = 0.15
**Generalized Anxiety Disorder Questionnaire** (GAD, 7-item)	6.51 (5.24)	7.97 (5.54)	*z* = −2.54, *p* = 0.01**
**Ethnicity**			*χ*^2^(3) = 2.56, *p* = 0.52
White/Caucasian	78.33% (*n* = 94)	83.09% (*n* = 290)	
Black	1.66% (*n* = 2)	2% (*n* = 7)	
Asian	5% (*n* = 6)	4.87% (*n* = 17)	
Other	15% (*n* = 18)	10.02% (*n* = 35)	
**Psychiatric disorders**	16% (*n* = 20)	27.75% (*n* = 96)	*χ*^2^(1) = 5.60, *p* = 0.02
Mood disorders (Bipolar disorders, Major depressive disorders)	3.20% (*n* = 4)	2.20% (*n* = 8)	*χ*^2^(1) = 2.43, *p* = 0.12
Anxiety disorders (Panic disorders, General anxiety disorders, Obsessive compulsive disorders, Post-traumatic stress disorders)	4.80% (*n* = 6)	10.10% (*n* = 37)	*χ* ^2^(1) = 0.52, *p* = 0.47
Mood and anxiety disorders	3.20% (*n* = 4)	8.79% (*n* = 30)	*χ* ^2^(1) = 1.01, *p* = 0.315
Other (e.g., could be a combination of a mood/anxiety disorder with another type of disorder such as eating disorder)	3.20% (*n* = 4)	4.67% (*n* = 17)	*χ* ^2^(1) = 0.06, *p* = 0.809
Not specified	1.6% (*n* = 2)	1.10% (*n* = 4)	*χ* ^2^(1) = 1.29, *p* = 0.256

To assess confusion between facial expressions, errors (i.e., the number of times when the actual expressed emotion was misclassified as one of the other emotions) were summed across participants and presented in confusion matrices. Sign *t*-tests (due to normality violation) were used to compare errors under masked vs. unmasked condition (i.e., to compare the number of times an emotion was misclassified as another in the masked vs. unmasked condition). Errors between males and females were assessed using a Mann-U Whitney test (effect sizes reported). Significance was *p* < 0.05.

Multivariate regression analyses were conducted to assess whether RT, accuracy, or combined performance scores were predicted by sex, age, stress, anxiety, depression, and loneliness scores. Independence of errors was tested using Durbin-Watson tests, homoscedasticity with scatterplots, multicollinearity using variance inflation factor values (i.e., values <10), and normality of residuals with Shapiro–Wilk tests. Logarithmic transformations were performed when normality was violated. Significance was *p* < 0.01 for regressions.

Finally, moderation analyses were performed to examine the influence of perceived stress, anxiety, depression and loneliness scores on relations between sex and RT, accuracy or performance scores (*p* < 0.05). Age was included as a covariate if it had a significant effect (*p* < 0.05) on the dependent variable or relationship.

For clarity and conciseness, and considering the numerous analyses and variables used in this study, only significant results are reported.

Sample size calculations indicated that with a rmAN(C)OVA [effect size = 0.2, alpha = 0.05 power = 0.95, 2 within-group measures (condition: masked/unmasked), correlation among repeated measures = 0.50], *N* = 84 would be required to yield differences between masked vs. unmasked faces on accuracy. To detect sex differences (as above), the total sample size required is *N* = 246. For multiple regressions, with power = 0.80 and a small effect size, *N* = 387 would be required.^1^ Therefore, we recruited >*N* = 400 participants.

## 3. Results

### 3.1. Demographics

Participant characteristics (120 M; 349F) are presented in Table 1. Males and females did not differ in age, education, gender (cisgender majority), or ethnicity (White majority) nor loneliness and depression scores. Females had higher stress (*p* < 0.001) and anxiety scores (*p* = 0.01), and reported more diagnosed psychiatric disorders than males (*p* = 0.02).

### 3.2. Repeated measures analyses of covariance (rmANCOVA)

Descriptive statistics for response times (RT) and accuracy are presented in the Supplementary material.

#### 3.2.1. Response times (RT)

**Figure 2:**
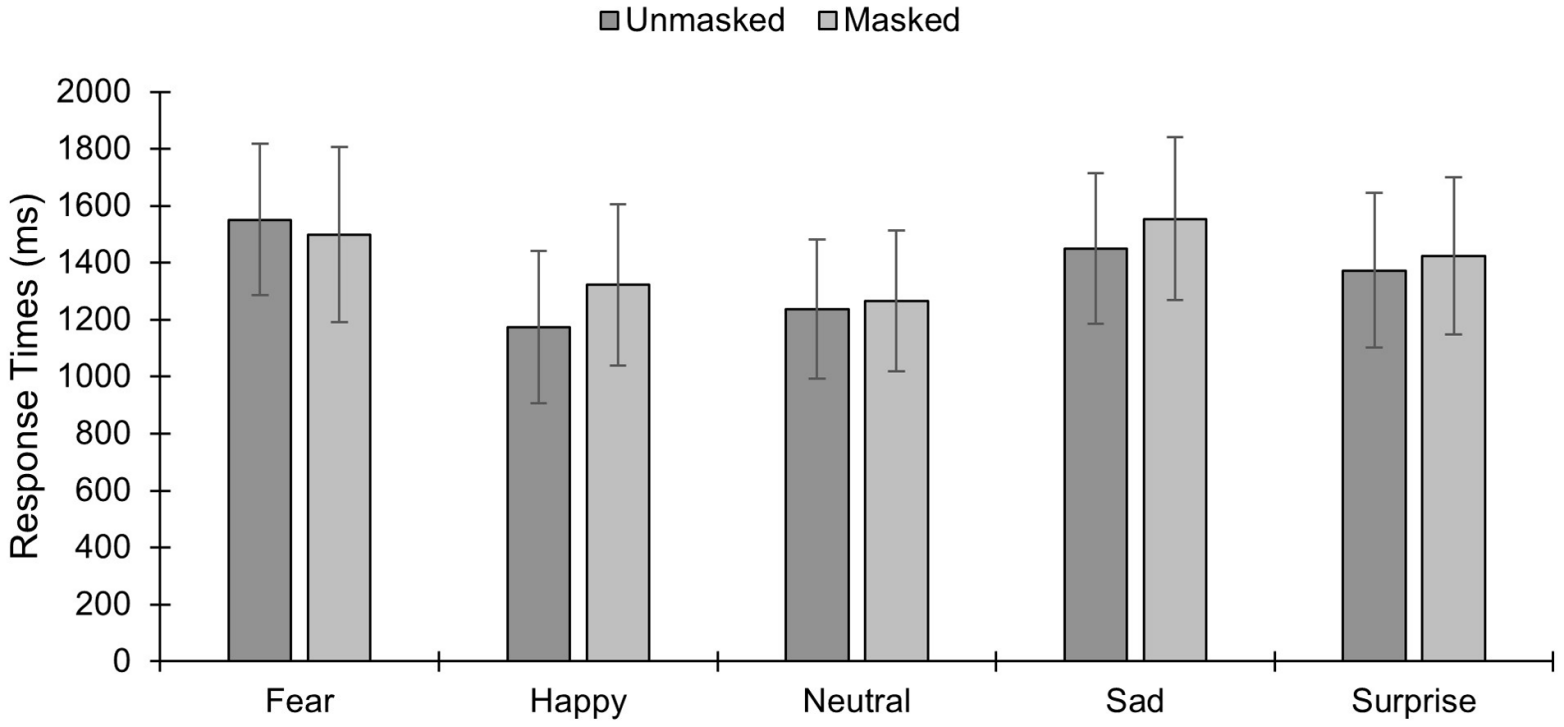
Reaction times (RT) to different facial expressions of emotion under masked and unmasked conditions (mean and standard deviations are presented). *p < 0.05; **p < 0.01; ***p < 0.001.

In total, *N* = 107 M/338F were used for the RT analyses (*N* = 23 participants did not answer correctly to all presentations of one or more emotions). A main effect of emotion existed [*F*(3.53,1579.17) = 61.16, *p* < 0.001, $\eta_{p}^{2}$ =0.12; Figure 2], and post-hoc Bonferroni-adjusted comparisons showed that RT was longer for fear vs. happy, neutral and surprise (*p*s < 0.001). RT was shorter for happy vs. sad and surprised (*p*s < 0.001). RT for sad vs. surprised was longer (*p* = 0.002), while it was shorter for neutral vs. sad and surprised faces (*p*s < 0.001). A main effect of sex existed [*F*(1,442) = 5.17, *p* = 0.02, $\eta_{p}^{2}$ =0.01], with shorter RT in females vs. males. Although no significant main effect of condition was observed at our significance threshold level [F(1,442) = 3.77, *p* = 0.05, $\eta_{p}^{2}$ =0.008], a condition×emotion interaction existed [*F*(3.49,1541.45) = 12.79, *p* < 0.001, $\eta_{p}^{2}$ =0.03], with longer RT for masked vs. unmasked expressions of happiness, sadness and surprise (*p*s < 0.001). Curiously, RT to fearful faces was longer for the unmasked vs. masked condition (*p* = 0.01). No significant difference in RT existed between masked vs. unmasked neutral faces (*p* = 0.06). Finally, results showed no sex×condition×emotion interaction [F(3.49,1541.45) = 2.47, *p* = 0.051, $\eta_{p}^{2}$ =0.006].

#### 3.2.2. Accuracy

**Figure 3:**
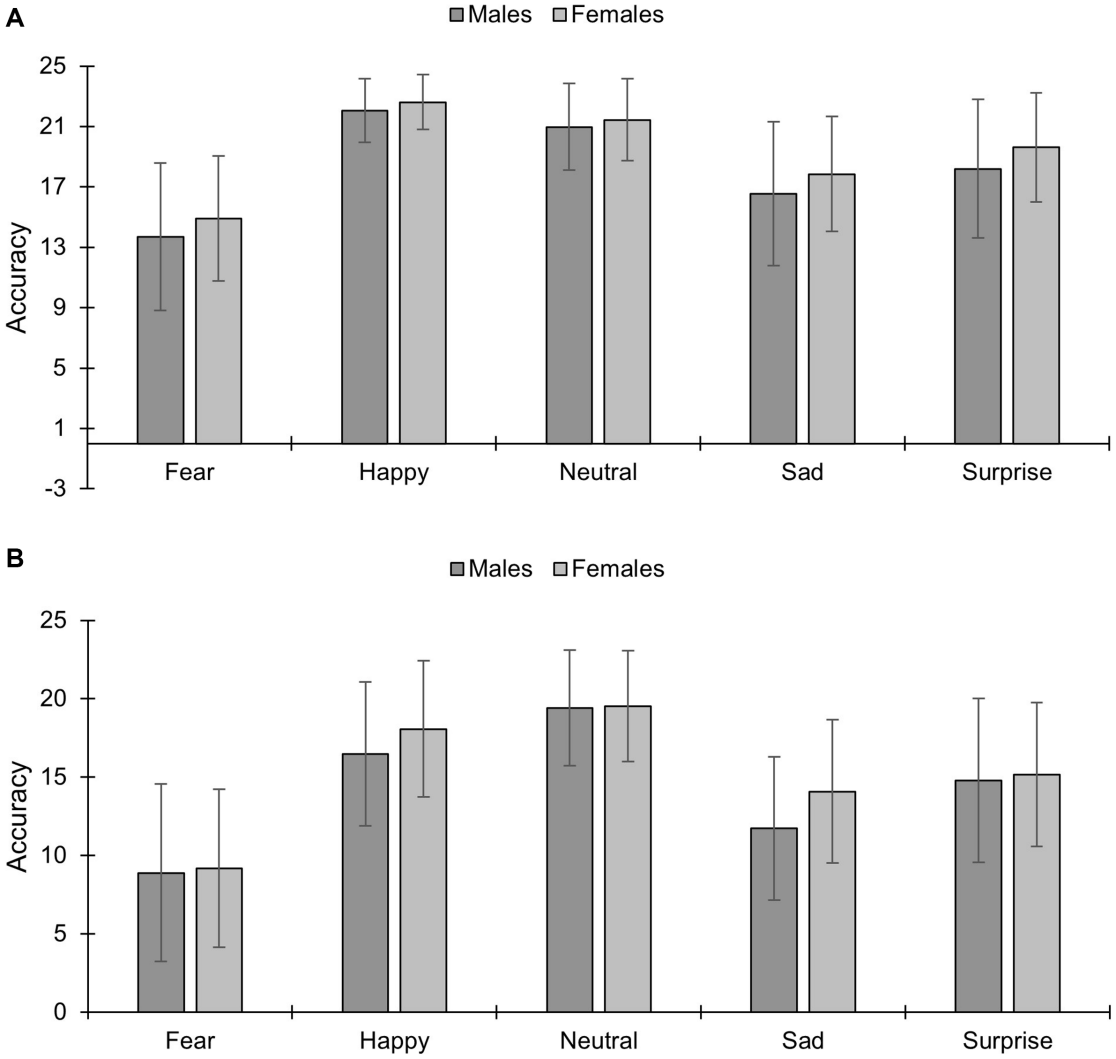
Accuracy to different facial expressions of emotion under **(A)** unmasked and **(B)** masked conditions in males and females (mean and standard deviations are presented). *p < 0.05; **p < 0.01; ***p < 0.001. The maximum accuracy score is 24 per emotion.

A main effect of condition emerged [*F*(1,466) = 123.35, *p* < 0.001, $\eta_{p}^{2}$ =0.21], with higher accuracy for unmasked (18.79 [maximum accuracy = 24], SEM = 0.01) vs. masked conditions (14.73, SME = 0.14). A main effect of emotion existed [*F*(2.66,1,237) = 98.84, *p* < 0.001, $\eta_{p}^{2}$ =0.18], with decreased accuracy for fear (11.67, SME = 0.21) vs. all other emotions (*p* < 0.001); accuracy for sadness (15.05, SME = 0.19) was lower than for surprise (16.94, SME = 0.19), and accuracy for both sadness and surprise were worse than for neutral (20.35, SME = 0.14) and happiness (19.81, SME = 0.14) (*p*s < 0.001). Finally, accuracy was higher for neutral vs. happy faces (*p* = 0.01). A main effect of sex existed [F(1,466) = 20.58, *p* < 0.001, $\eta_{p}^{2}$ =0.04], with females (17.24, SEM = 0.11) having higher accuracy than males (16.28, SEM = 0.18). A sex×emotion interaction existed [*F*(4,466) = 3.13, *p* = 0.01, $\eta_{p}^{2}$ =0.007]. Bonferroni-adjusted post-hocs showed that females vs. males had higher accuracy for happiness (*p* < 0.001), sadness (*p* < 0.001) and surprise (*p* < 0.03). A condition×emotion interaction existed [*F*(3.54,1649.42) = 8.98, *p* < 0.001, $\eta_{p}^{2}$ =0.02]; its breakdown revealed that accuracy was higher for unmasked vs. masked expressions for all emotions (*p*s < 0.001). Finally, a condition×emotion×sex interaction existed [*F*(4, 420) = 6.20, *p* < 0.001, $\eta_{p}^{2}$ =0.02; Figure 3]. For unmasked expressions, Bonferroni-adjusted analyses showed that females were more accurate than males in identifying fearful (*p* < 0.01), happy (*p* = 0.006), sad (*p* = 0.002), surprise (*p* < 0.001), but not neutral faces (*p* = 0.11). For masked faces, females vs. males were more accurate in recognizing happy and sad expressions (*p* < 0.001), but not fear (*p* = 0.62), surprise (*p* = 0.49) or neutral faces (*p* = 0.78).

### 3.3. Confusion matrices

Misattribution errors were analysed using confusion matrices (Supplementary Tables S2–S7). For both masked/unmasked conditions, most misattributions errors occurred when participants incorrectly identified fearful faces as surprised ones. Other common misattributions in both conditions were when a surprised or sad face was perceived as a fearful one. Under masked conditions, happy, sad and surprised faces were also commonly mistaken for neutral ones. Misattribution errors were more numerous in the masked vs. unmasked condition (Table 2).

**Table 2:** Confusion matrix of sign *t*-test analyses of expressed and perceived facial expressions of emotion under masked vs. unmasked conditions.

	**Perceived**								
**Expressed**	**Emotion**	**Fear**	**Happy**	**Neutral**	**Sad**	**Surprise**			
	**Fear**		*Z* = −6.79***	*Z* = −1.22 ns	*Z* = −14.30***	*Z* = −19.73***			
			*r* = 0.31	*r* = 0.06	*r* = 0.66	*r* = 0.91			
	**Happy**	*Z* = −4.25***		*Z* = −19.62***	*Z* = −4.34***	*Z* = −4.83***	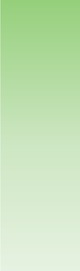	≥0.50 – Large effect size	
		*r* = 0.19		*r* = 0.91	*r* = 0.20	*r* = 0.22			
	**Neutral**	*Z* = −6.57***	*Z* = −2.38*		*Z* = −11.04***	*Z* = −0.65 ns		<0.50 – Medium effect size	
		*r* = 0.30	*r* = 0.11		*r* = 0.51	*r* = 0.03			
	**Sad**	*Z* = −2.62*	*Z* = −7.96***	*Z* = −17.35***		*Z* = −4.27***		<0.30 – Small effect size	
		*r* = 0.12	*r* = 0.37	*r* = 0.80		*r* = 0.20			
	**Surprise**	*Z* = −8.49***	*Z* = −5.26***	*Z* = −15.83***	*Z* = −2.98**				
		*r* = 0.39	*r* = 0.24	*r* = 0.73	*r* = 0.14				

ns, non significant; *p < 0.05; **p < 0.01; ***p < 0.001.

Mann–Whitney U tests were used to assess sex differences in confusion errors (Tables 3, 4). For the unmasked condition, errors were significantly more common in males vs. females for happy faces perceived as fearful, neutral faces perceived as surprised ones and sad faces perceived as happy ones. Under masked conditions, confusion errors were significantly more numerous in males vs. females for happy faces mistaken with neutral, sad and surprised ones. Finally, males vs. females were more likely to mistake neutral faces with those expressing fear and sadness; sad faces were also mistaken with neutral and surprise more frequently by males vs. females.

**Table 3:** Confusion matrix of Mann–Whitney analyses of expressed and perceived facial expressions of emotion under unmasked conditions between males vs. females.

	**Perceived**								
**Expressed**	**Emotion**	**Fear**	**Happy**	**Neutral**	**Sad**	**Surprise**			
	**Fear**		*Z* = −0.94 ns	*Z* = −1.88 ns	*Z* = −0.39 ns	*Z* = −0.40 ns			
			*r* = 0.04	*r* = 0.09	*r* = 0.02	*r* = 0.02			
	**Happy**	*Z* = −2.73**		*Z* = −1.59 ns	*Z* = −0.84 ns	*Z* = −1.25 ns	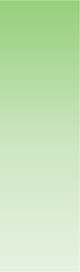	≥0.50 – Large effect size	
		*r* = 0.13		*r* = 0.07	*r* = 0.04	*r* = 0.06			
	**Neutral**	*Z* = −0.31 ns	*Z* = −0.02 ns		*Z* = −0.64 ns	*Z* = −2.04*		<0.50 – Medium effect size	
		*r* = 0.01	*r* = 0.001		*r* = 0.03	*r* = 0.09			
	**Sad**	*Z* = −1.46 ns	*Z* = −2.33*	*Z* = −1.21 ns		*Z* = −1.36 ns		<0.30 – Small effect size	
		*r* = 0.07	*r* = 0.11	*r* = 0.06		*r* = 0.06			
	**Surprise**	*Z* = −1.96 ns	*Z* = −0.81 ns	*Z* = −0.87 ns	*Z* = −1.51 ns				
		*r* = 0.09	*r* = 0.04	*r* = 0.04	*r* = 0.07				

ns, non significant; *p < 0.05; **p < 0.01; ***p < 0.001.

**Table 4:** Confusion matrix of Mann–Whitney analyses of expressed and perceived facial expression of emotion under masked conditions between males vs. females.

	**Perceived**								
**Expressed**	**Emotion**	**Fear**	**Happy**	**Neutral**	**Sad**	**Surprise**			
	**Fear**		*Z* = −1.71 ns	*Z* = −0.96 ns	*Z* = −0.53 ns	*Z* = −0.02 ns			
			*r* = 0.08	*r* = 0.04	*r* = 0.02	*r* = 0.001			
	**Happy**	*Z* = −0.37 ns		*Z* = −2.98**	*Z* = −1.98*	*Z* = −3.60***	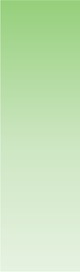	≥0.50 – Large effect size	
		*r* = 0.02		*r* = 0.14	*r* = 0.09	*r* = 0.17			
	**Neutral**	*Z* = −3.13**	*Z* = −0.87 ns		*Z* = −2.22*	*Z* = −0.24 ns		<0.50 – Medium effect size	
		*r* = 0.14	*r* = 0.04		*r* = 0.10	*r* = 0.01			
	**Sad**	*Z* = −0.44 ns	*Z* = −1.44 ns	*Z* = −4.32***		*Z* = −3.19**		<0.30 – Small effect size	
		*r* = 0.02	*r* = 0.07	*r* = 0.20		*r* = 0.15			
	**Surprise**	*Z* = −0.53 ns	*Z* = −1.02 ns	*Z* = −0.62 ns	*Z* = −0.20 ns				
		*r* = 0.02	*r* = 0.05	*r* = 0.03	*r* = 0.01				

ns, non significant; *p < 0.05; **p < 0.01; ***p < 0.001.

### 3.4. Regression analyses

#### 3.4.1. Unmasked conditions

Regression results are in Supplementary Table S8. For average RT (i.e., all emotional expressions), the overall model was significant [*R*^2^ = 0.14, *F*(6,469) = 12.77, *p* < 0.001]. Age was the strongest predictor of RT (*t* = 7.95, *B* = 76.03, *p* < 0.001) with higher age predicting longer RT. ULS (loneliness) scores were the second strongest predictor of average RT (*t* = −2.26, *B* = −24.90, *p* = 0.02); higher ULS scores were associated with shorter RT. Finally, sex was also a predictor of average RT (*t* = −1.99, *B* = −18.27, *p* = 0.05), with longer RT associated with being male.

The model for RT to *happy* faces was significant [*R*^2^ = 0.12, *F*(6,468) = 10.29, *p* < 0.001], with age as a positive predictor (*t* = 6.73, *B* = 0.03, *p* < 0.001) and being male as a predictor of longer RT (*t* = −2.52, *B* = −0.01, *p* = 0.01). ULS scores were also a predictor (*t* = −1.97, *B* = −0.01, *p* = 0.05), with higher ULS scores predicting shorter RT to happy faces. For RT to *sad* faces, the model was significant [*R*^2^ = 0.06, *F*(6,468) = 4.52, *p* < 0.001]; age was the strongest predictor (*t* = 4.46, *B* = 0.02, *p* < 0.001), followed by ULS (*t* = −2.08, *B* = −0.01, *p* = 04), indicating that a longer RT to sad faces were predicted by an older age and lower loneliness scores. The model for RT to *fearful* faces was significant [*R*^2^ = 0.12, F(6,469) = 10.32, *p* < 0.001], with older age (*t* = 7.00, *B* = 0.03, *p* < 0.001) and being male (*t* = −2.68, *B* = −0.01, *p* = 0.01) predicting longer RT. Finally, for RT to *neutral* and *surprised* faces, the models were significant [neutral: *R*^2^ = 0.09, *F*(6,468) = 7.50, *p* < 0.001; surprise: *R*^2^ = 0.12, *F*(6,466) = 10.8, *p* < 0.001]; age as the only significant positive predictor (neutral: *t* = 6.35, *B* = 0.03, *p* < 0.001; surprise: *t* = 7.00, *B* = 87.80, *p* < 0.001). PSS, GAD and PHQ scores were not predictors of RT for any emotions.

For average accuracy (i.e., all expressions), a violation of normality for the residuals existed even after ln-transformation; thus, analyses are presented for non-transformed data. The model was significant [*R*^2^ = 0.05, F(6,468) = 3.97, *p* < 0.001], but sex was the only significant predictor, with higher accuracy predicted by being female (*t* = 4.64, *B* = 2.21, *p* < 0.001). A violation of normality for the residuals existed for accuracy to *neutral* and *sad* faces, even after a transformation (non-transformed data presented). For accuracy to *fear, neutral*, *sad* and *surprised* faces, models were significant [fear: *R*^2^ = 0.05, *F*(6,468) = 3.82, *p* < 0.001; neutral: *R*^2^ = 0.04, F(6,468) = 3.33, *p* = 0.003; sad: *R*^2^ = 0.05, F(6,468) = 4.02, *p* < 0.001; surprise: *R*^2^ = 0.05, F(6,468) = 3.65, *p* < 0.001]. Sex was a predictor for most models, with being female as a predictive of better accuracy to *fearful*, *sad* and *surprised* faces (fear: *t* = 3.97, *B* = 0.03, *p* < 0.001; sad: *t* = 3.26, *B* = 0.62, *p* = 0.001; surprise: *t* = 3.19, *B* = 0.02, *p* = 0.002). Greater age predicted lower accuracy to *fear* (*t* = −2.45, *B* = −0.021, *p* = 0.02) and *neutral* (*t* = −3.61, *B* = −0.48, *p* > 0.001), yet higher accuracy to *sad* faces (*t* = 2.74, *B* = 0.54, *p* = 0.006). None of the mental health scores were significant predictors of accuracy to unmasked emotional or neutral faces.

#### 3.4.2. Masked conditions

Regressions are presented in Supplementary Table S9. For average RT, the model was significant [*R*^2^ = 0.14, *F*(6,468) = 13.25, *p* < 0.001], with greater age as the strongest predictor of longer RT (*t* = 8.38, *B* = 82.45, *p* < 0.001), followed by being male (*t* = −1.99, *B* = −18.79, *p* = 0.05). For both *happy* and *sad* faces, the models were significant [happy: *R*^2^ = 0.12, *F*(6,467) = 10. 40, *p* < 0.001; sad: *R*^2^ = 0.10, *F*(6,464) = 8.29, *p* < 0.001]; age was a positive predictor of RT (happy: *t* = 7.19, *B* = 0.03, *p* < 0.001; sad: *t* = 6.08, *B* = 0.02, *p* < 0.001), and being male predicted longer RT (happy: *t* = −2.78, *B* = −0.01, *p* = 0.006; sad: *t* = −3.31, *B* = −0.01, *p* = 0.001). Similarly, models were significant for RT to *fear*, *neutral* and *surprised* faces [fear: *R*^2^ = 0.05, *F*(6,453) = 4.15, *p* < 0.001; neutral: *R*^2^ = 0.10, F(6,468) = 8.12, *p* < 0.001; surprise: *R*^2^ = 0.12, *F*(6,466) = 10.80, *p* < 0.001], with age as the only positive predictor (fear: *t* = 4.57, *B* = 0.02, *p* < 0.001; neutral: *t* = 6.70, *B* = 0.03, *p* < 0.001; surprise: *t* = 6.67, *B* = 87.61, *p* < 0.001). Mental health scores were not predictors of RT to emotional or neutral masked faces.

The model for average accuracy was significant [*R*^2^ = 0.05, F(6,468) = 3.60, *p* = 0.002], with greater age (*t* = −3.37, *B* = −0.02, *p* = 0.001) and being male (*t* = 3.11, *B* = 0.01, *p* = 0.002) predicting lower accuracy. The assumption of normality for the residuals in the regressions on accuracy were violated for fearful faces, even with ln-transformation (analyses presented for non-transformed data). The model was significant [*R*^2^ = 0.03, F(6,468) = 2.70, *p* = 0.01], with older age and higher PSS scores predicting lower accuracy to *fearful* faces (age: *t* = −2.66, *B* = −0.67, *p* = 0.008; PSS: *t* = −2.17, *B* = −0.81, *p* = 0.03). For *sad* faces, the overall model was significant [*R*^2^ = 0.05, F(6,464) = 3.96, *p* = 0.001], with being female (*t* = 3.94, *B* = 0.04, *p* < 0.001) predicting better accuracy. Loneliness, anxiety, and depression scores were not significant predictors of accuracy to any of the emotional or neutral masked faces.

### 3.5. Moderation analyses

**Figure 4:**
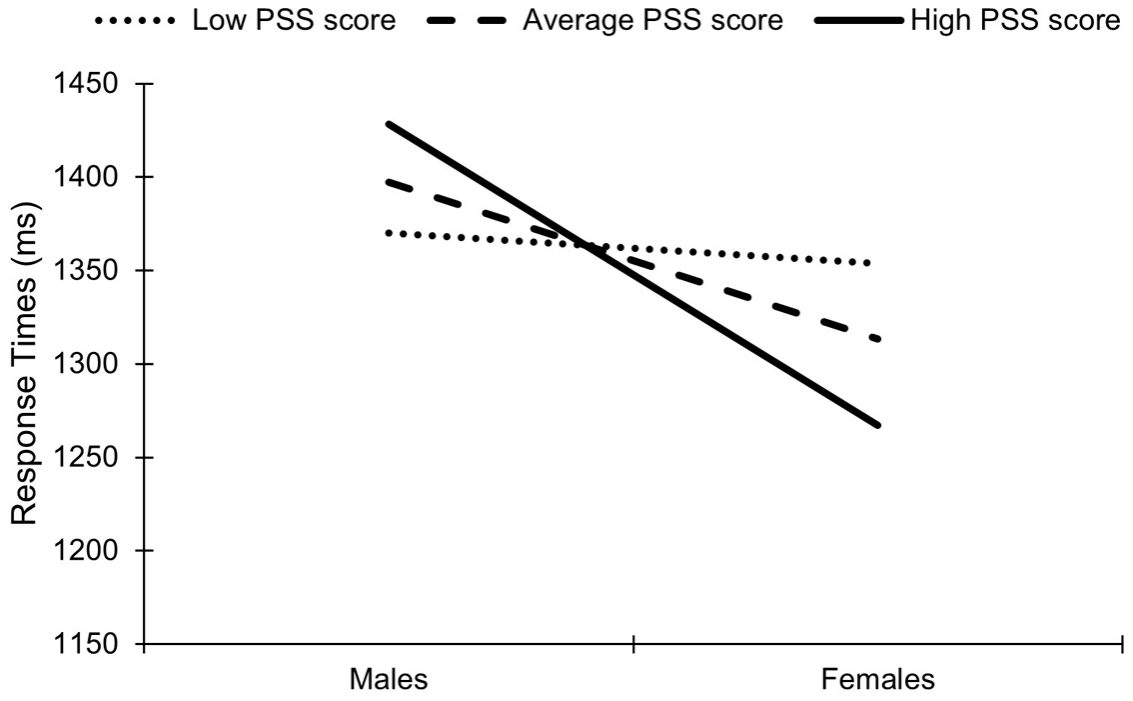
The moderating role of Perceived Stress Scale (PSS)-indexed stress scores on the relationship between sex and response times to masked happy faces. The low, average, and high scores correspond to the 16th, 50th, and 84th percentile respectively.

Moderation analyses were conducted to examine whether the relationships between accuracy, RT, performance, and sex were moderated by mental wellness (PHQ-9, ULS, PSS scores). Shapiro–Wilk tests revealed a violation of normality for residuals. The overall model was significant regarding the influence of PSS scores on the relationship between sex and RT for masked happy faces [*R*^2^ = 0.32, *F*(4,464) = 5.07, *p* = 0.002]. As represented in Figure 4, a sex×PSS score interaction existed on RT to masked happy faces [*R*^2^ = 0.11, *F*(1,464) = 5.16, *p* = 0.02]; males with high PSS scores had a longer RT to masked happy faces than males with average and low scores. In contrast, females with high PSS scores had a shorter RT to masked happy faces than males with average and low scores. The conditional effects were not significant for low PSS score (*B* = −16.31, *p* = 0.68) but were significant for average (*B* = −83.82, *p* = 0.007) and high PSS scores (*B* = −160.98, *p* = 0.001). This indicates that the effects of PSS score on the relationship between sex and RT to masked happy faces is stronger for higher levels of perceived stress scores.

## 4. Discussion

In this study, we replicated the influence of masks and sex on facial expression recognition. Most novel, we investigated the effects of sex on misattribution errors, and the influence of loneliness, stress, anxiety, depression symptoms on facial expression recognition under masked/unmasked conditions. Expectedly, facial expression recognition was impaired by facial covering (i.e., decreased accuracy/longer RT), and females showed an advantage vs. males (i.e., higher accuracy/less misattribution errors), confirming our first hypothesis. Interestingly and contrasting from our second hypothesis, loneliness scores and perceived stress scores predicted the successful recognition of some emotions and their effect differed depending on the respondent’s sex. Facial emotion recognition was not associated with anxiety nor depression symptoms in our cohort.

Our results confirm that facial expression recognition is impaired with lower face occlusion (Carbon, 2020; Gori et al., 2021; McCrackin et al., 2022), particularly for happiness, sadness and surprise. However, masks did not have the same effect on all emotional faces. For instance, RT to fearful faces were *longer* for unmasked vs. masked expressions, although accuracy was significantly lower (near chance). Upper facial features are particularly important in recognizing fearful faces (Wegrzyn et al., 2017), thus, lower face occlusion could yield faster responses as there might be fewer spatial distractions, albeit compromising accuracy. Under both masked/unmasked conditions, most misattributions errors occurred when participants confused fearful and surprised faces; this is consistent with the literature as both expressions are characterized by widened eyes (Wegrzyn et al., 2017), and must therefore rely on more granular assessment of other facial cues. Other common misattributions arose between sad and fearful faces, which have similar negative emotional valence, and may therefore be more often confused than with emotions of opposing valance (Marini et al., 2021).

Overall, females vs. males were faster at correctly identifying emotions under both conditions and were more accurate in recognising unmasked expressions of fear, as well as masked and unmasked expressions of happiness, and sadness. Additionally, misattribution errors were more common in males vs. females for both conditions, though more so with facial occlusion. Sex was an important predictor of superior facial expression recognition (i.e., better accuracy, lower RT, better composite performance). These results are consistent with previous research (Grundmann et al., 2021; Ramachandra and Longacre, 2022), and could be linked to gender based socialisation (i.e., girls might be more encouraged to express and identify emotions than boys) and stereotypes (Rodriguez-Gomez et al., 2020). This, combined with hormonal differences, could underlie (or be accounted for by) differential brain activity in males vs. females in areas such as the amygdala, the superior frontal gyrus or the middle temporal gyrus during the processing of emotional faces (Jenkins et al., 2018).

Also consistent with the literature (Hayes et al., 2020), older age predicted longer RT for all emotions, and lower accuracy for fear (regardless of condition). This could be accounted for by age-related brain changes, perhaps reflecting less efficient neuronal processing (Turner et al., 2020). Using a keyboard/computer might also have exacerbated functional (e.g., degradation in motor skills, poorer eyesight) and cognitive impairments (e.g., decline working memory or cognitive control abilities), and performance might have been affected by more negative attitudes towards technology more typical in older people (Sonderegger et al., 2016). Finally, the data set used in our task did not vary in terms of age of stimuli/actors; previous research found higher accuracy when using same aged stimuli in younger and older adults (Ferreira et al., 2021). As such, the age of the experimental stimuli might reduce the impact of the participant’s age on performance.

Intriguingly, higher perceived stress scores predicted lower accuracy to masked fearful faces. This might suggest that higher stress is associated with greater avoidance and/or poorer processing of more ambiguous yet potentially threatening faces (i.e., masked fearful faces). This contrasts somewhat with previous literature showing increased accuracy to multiple emotions, including fearful faces, following an acute stress, perhaps reflecting a priming effect (Daudelin-Peltier et al., 2017; Barel and Cohen, 2018; Domes and Zimmer, 2019). However, the PSS scale measures the degree to which life situations are appraised as stressful and the level of currently experienced stress. In other words, it taps into more chronic stress perception and personality features (vs. a response to a specific trigger); this could explain these literature differences. Further, perceived stress was higher for females than males and influenced the relationship between sex and RT to masked happy faces. Specifically, while high perceived stress scores were linked to longer RT to masked happy faces for males, the results were opposite in females. This hints at the possibility that higher baseline stress affects facial processing differently in the two sexes; in females, it appears that heightened (though not necessarily pathological) stress levels may aid in assessing positively valanced expressions (i.e., a non-threatening cue). Conversely, higher perceived stress in males might be associated with longer processing of a potentially mood-incongruent (and/or rather unambiguous) facial expression. However, this should be further investigated in future research. Sex differences in perceived stress have previously been noted (Graves et al., 2021), although not in relation to facial emotional processing. Neuroimaging studies have revealed differential brain activity between the sexes while viewing angry facial expressions following acute stress induction (Mather et al., 2010), which also suggests a distinct influence of stressors on neural correlates of emotional processing in males and females.

Higher loneliness scores predicted shorter RT (but not lower accuracy) to unmasked faces overall, and particularly for happy and sad unmasked faces. This supports previous work reporting that lonelier people have increased sensitivity to sad facial expressions (Vanhalst et al., 2017), and is somewhat in line with the mood-congruent processing hypothesis typically ascribed to depression (Schmid and Schmid Mast, 2010).

Interestingly, sex was not a moderator of the relationship between mental wellness and emotion recognition. Nevertheless, although we found no sex differences in reported loneliness, loneliness scores moderated the influence of sex on composite performance of unmasked sad faces (Supplementary material). Specifically, high loneliness was linked to better performance to unmasked sad faces for males, which was not the case for females. This suggests that lonely males might be more sensitive to recognizing facial expressions of a negative valence (which, to a certain extent might be consistent with a negative processing bias (Hawkley and Cacioppo, 2010)), while loneliness scores do not appear to have much influence on facial recognition performance in females (perhaps due ‘ceiling like’ performance levels, regardless of loneliness). It is feasible that individuals pre-selected for extreme loneliness scores (e.g., top/bottom quartiles) might show more pronounced behavioural differences. However, this is speculative, and warrants follow-up with more research.

Anxiety symptoms were significantly higher in females. However, these symptoms were not associated with facial emotion recognition; the same was true for depressive symptoms. Granted, mean anxiety and depression symptoms scores were low-to-moderate in our sample which could account for our null findings, and are consistent with others’ (Cooper et al., 2008). Different tasks (e.g., those relying not just on responses but aspects such as eye-tracking) might enable the assessment of attention towards specific emotions in a manner that could reveal more granular relations with depression and anxiety symptoms. This should be considered in future work.

## 5. Limitations and conclusions

While this study was the first to assess the influence of mental wellbeing on facial emotion recognition, replicates and builds upon previous work in smaller samples, certain limitations exist. First, as is a typical limitation of online studies, we could not control the experimental setting (e.g., lightning, distraction, instruction comprehension), which could have contributed to data variability. On the other hand, the naturalistic nature of such study designs, coupled with the large sample, generally have greater ecological validity than in-laboratory assessments. Further, we used the Karolinska Sleepiness Scale (KSS, 1-item) and asked participant to rate their engagement with the tasks; initial correlation analyses between these scales and RTs or accuracy measures did not reveal any significant results (data not shown). Thus, participants feelings of sleepiness and engagement did not seem to be significantly associated to performance. Second, our task consisted of static images expressing rather exaggerated emotions, thus decreasing ecological validity. Future research could consider using stimuli expressing subtle emotions and dynamic faces, which have not been extensively studied in the context of facial occlusion effects. Third, our sample had a large proportion of females (which is common in similar online studies) (Grundmann et al., 2021; Carbon et al., 2022; Proverbio and Cerri, 2022); which might have impacted statistical analyses. Our reported sex differences could have also been influenced by menstrual cycle phase or hormonal contraceptive use. For instance, previous results revealed increased accuracy during the follicular phase, when progesterone levels are low and estradiol levels are high, and reduced performance with hormonal contraceptive use (Osório et al., 2018). We did not collect data on this as part of the current study but recommend doing so in the future. Finally, because the data was collected during the COVID-19 pandemic (though not during the very initial stages of the pandemic), it is possible that this context influenced responses, particularly regarding mental health symptoms and the identification of emotions on masked faces. However, it is unlikely that the data would substantially change when collected outside this timeframe.

To conclude, this study confirmed the influence of facial occlusion on facial expression recognition. Further, this study highlights the importance of considering sex and mental wellness as predictors of emotion recognition. Such insight could be valuable when considering policies involving mask-wearing in potential future pandemics. Additionally, our data corroborate the potentially adverse effects of mask-wearing on communication (e.g., ability to identify another individual as threatening could be impaired), which likely has implications in health care settings, for instance, as effective facial expression skills could be determinant in providing effective care (e.g., non-verbal cues might not be efficiently processed/misunderstood).

## Data availability statement

The raw data supporting the conclusions of this article will be made available by the authors, without undue reservation.

## Ethics statement

The studies involving humans were approved by the Research Ethics Boards of the ROMHC, St. Francis Xavier University and Dawson Research Ethics Board (REB#s 2020024; 25316; JAWON2021181). The studies were conducted in accordance with the local legislation and institutional requirements. The participants provided their written informed consent to participate in this study.

## Author contributions

Each of the authors participated in this research by contributing to its conceptualization (NJ, SC, LB, and MH), methodology (NJ, SC, LB, and MH), formal analysis (MH, KB, and GA), and draft preparation (MH, KB, GA, and NJ) as well as reviewing and editing (SC, LB, KB, and NJ). All authors contributed to the article and approved the submitted version.

## Footnotes

^1^ https://www.danielsoper.com/statcalc/calculator.aspx?id=1
