# Supplementary material for: Recognition of masked and unmasked facial expressions in males and females and relations with mental wellness
Source: Front Psychol. 2023 Oct 30;14:1217736. doi: 10.3389/fpsyg.2023.1217736 (PMC10643509; doi:10.3389/fpsyg.2023.1217736)
Supplement: Supplementary file 1 [file Data_Sheet_1.docx]

**Supplementary Materials**

**NimStim Stimuli**

The following images were used for the practice trials: 02F_SP_O. 03F_HA_O. 11F_NE_C. 15F_HA_O. 17F_SA_C. 20M_NE_O. 36M_FE_O. 38M_SA_C. 39M_SP_O. 40M_FE_O.

The following images were used for the study trials: 09F. 13F. 18F. 19F. 28M. 37M. 42M. 43M: HA=4C(closed)/4O(open)mouths; NE=4O/4C; SP=8O; SA=6O/2C; FE=4O/4C.

**Supplementary Figure 1.** Instructions for the Emotional Processing Tasks


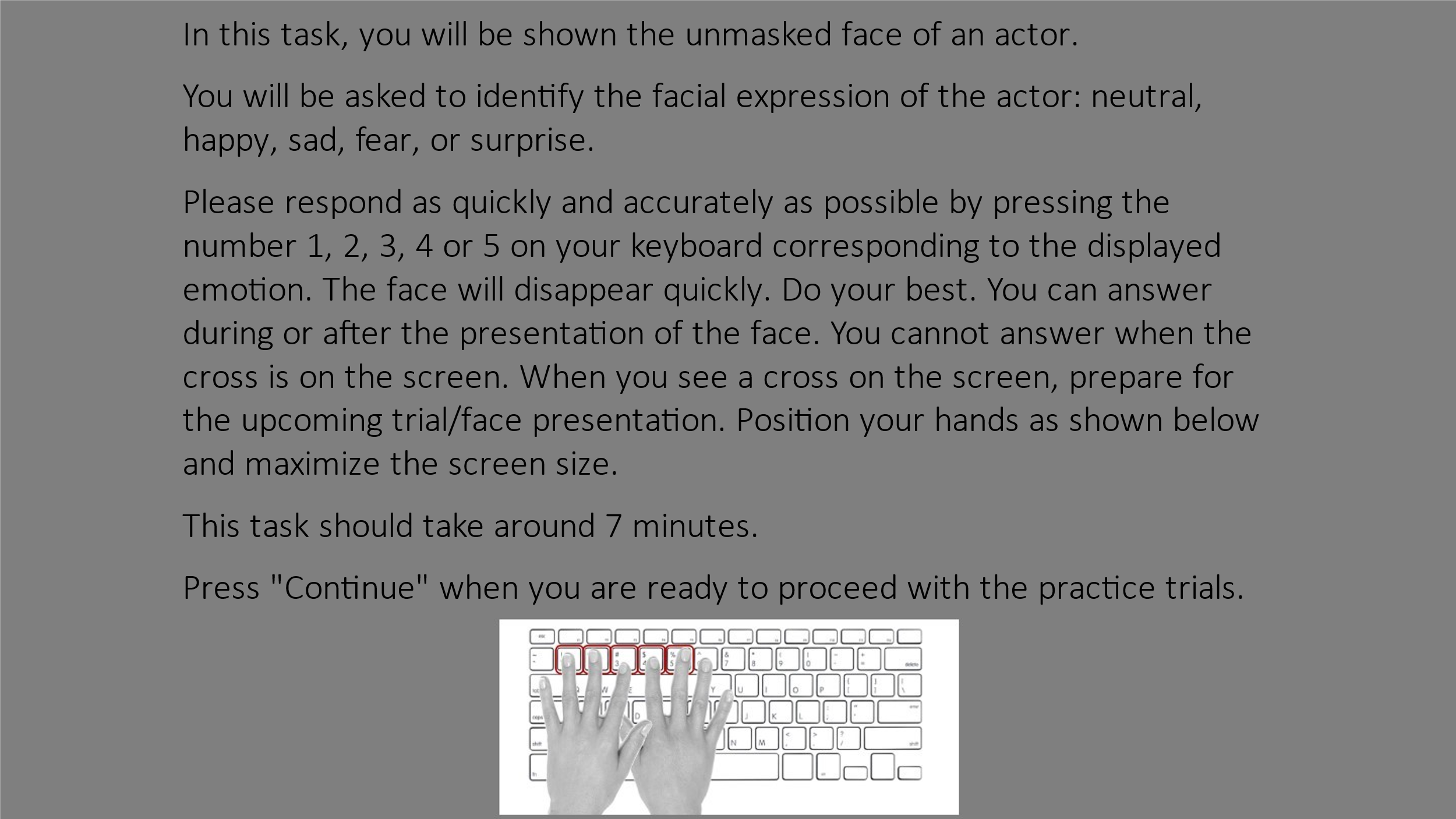


**Supplementary Table 1.** Descriptives statistics of response time (ms), and accuracy per emotion (means and standard deviation presented)

|  | **Response Time** | **Accuracy** |
| --- | --- | --- |
| **Unmasked Facial Expressions** |  |  |
| **Fear** | 1549.15 (270.03) | 14.59 (4.36) |
| **Happy** | 1176.20 (267.15) | 22.46 (1.90) |
| **Neutral** | 1237.65 (242.89) | 21.32 (2.76) |
| **Sad** | 1453.04 (264.20) | 17.51 (4.10) |
| **Surprise** | 1370.37 (270.82) | 19.25 (3.94) |
| **Masked Facial Expressions** |  |  |
| **Fear** | 1496.93 (308.66) | 9.10 (5.20) |
| **Happy** | 1328.68 (289.24) | 17.66 (4.46) |
| **Neutral** | 1267.55 (251.49) | 19.51 (3.57) |
| **Sad** | 1554.47 (291.44) | 13.48 (4.69) |
| **Surprise** | 1420.26 (283.99) | 15.05 (4.76) |

Note. The maximum accuracy score is 24 per emotion.

**Performance**

A repeated measures analyses of variance (rmANCOVA) revealed a main effect of sex [F(1,442)=12.64, *p*<.001, η^2^=03], with females vs. males having higher performance scores. A main effect of emotion existed [F(3.43,1517.59)=4.17, *p*=.004, η²=.01], though Bonferroni-adjusted pairwise comparisons showed no significant differences in performance scores between emotions. A sex×emotion interaction existed [F(3.43,1517.59)=2.64, *p*=.04, η²=.006], with post-hoc tests indicating that females vs. males had higher performance scores for happiness (*p*<.001), sadness (*p*<.001) and surprise (*p*<.02). A condition×emotion×sex interaction emerged [F(3.88,1714.58)=3.12, *p*=.01, η^2^=.007; **Supplementary Figure 1**]; focus was on between-sex comparisons. For the unmasked condition, females vs. males had higher for happiness (*p*=.004), sadness (*p*=.02), and surprise (*p*=.005). For the masked condition, females vs. males had higher performance for happiness (*p*=.001) and sadness (*p*<.001). Moderation analyses revealed a significant influence of loneliness scores on the relationship between sex and performance for unmasked sad faces [R²=.02, F(3,463)=4.42, p=.005]. A significant sex × loneliness score interaction existed on performance scores to unmasked sad faces [R²=.008, F(1,463) =3.95, p=.05]. This indicated that males with high loneliness scores had better performance to unmasked sad faces than males with average and low loneliness scores; performance was high in females, regardless of loneliness scores. Additionally, conditional effects were significant for low (B=.82, p<.001) and average loneliness scores (B=.40, p=.02), but not for high loneliness scores (B=.13, p=.59).

**Supplementary Figure 2.** Performance scores in males and females to all facial expression of emotion under **(A)** unmasked and **(B)** masked conditions.


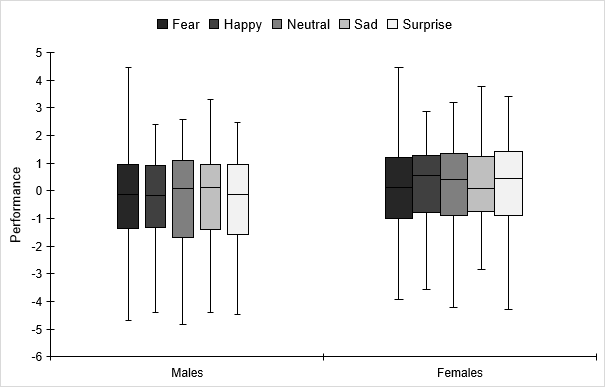


a


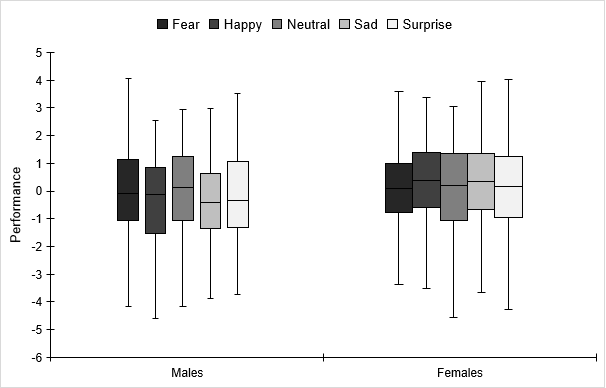


b

**Supplementary Table 2.** Confusion matrix of expressed and perceived facial expressions of emotions under unmasked condition collapsed across all participants (N=469) representing the percentage of misattribution errors.

| Error % | Perceived | | | | | |
| --- | --- | --- | --- | --- | --- | --- |
| Expressed | Emotion | Fear | Happy | Neutral | Sad | Surprise |
|  | Fear |  | .71% | 3.25% | 12.15% | 23.39% |
|  | Happy | .47% |  | 1.48% | 1.77% | 1.79% |
|  | Neutral | 1.42% | .78% |  | 5.77% | 1.76% |
|  | Sad | 16.15% | 1.10% | 6.00% |  | 2.24% |
|  | Surprise | 15.40% | 2.19% | 1.21% | 1.00% |  |

**Supplementary Table 3.** Confusion matrix of expressed and perceived facial expressions of emotions under masked condition collapsed across all participants (N=469) representing the percentage of misattribution errors.

| Error % | Perceived | | | | | |
| --- | --- | --- | --- | --- | --- | --- |
| Expressed | Emotion | Fear | Happy | Neutral | Sad | Surprise |
|  | Fear |  | 1.23% | 1.40% | 1.50% | 30.79% |
|  | Happy | .62% |  | 9.85% | 1.67% | 1.64% |
|  | Neutral | 1.55% | .78% |  | 5.96% | .95% |
|  | Sad | 6.98% | 1.84% | 11.84% |  | 1.59% |
|  | Surprise | 12.22% | 2.17% | 4.68% | .75% |  |

**Supplementary Table 4.** Confusion matrix of expressed and perceived unmasked facial expressions of emotions in females only (N=349) representing the percentage of misattribution errors.

| Error % | Perceived | | | | | |
| --- | --- | --- | --- | --- | --- | --- |
| Expressed | Emotion | Fear | Happy | Neutral | Sad | Surprise |
|  | Fear |  | .66% | 3.09% | 12.56% | 23.96% |
|  | Happy | .34% |  | 1.44% | 1.71% | 1.82% |
|  | Neutral | 1.47% | .83% |  | 5.80% | 1.69% |
|  | Sad | 16.28% | .93% | 6.32% |  | 2.20% |
|  | Surprise | 14.79% | 2.11% | 1.11% | .89% |  |

**Supplementary Table 5.** Confusion matrix of expressed and perceived masked facial expressions of emotions in females only (N=349) representing the percentage of misattribution errors.

| Error % | Perceived | | | | | |
| --- | --- | --- | --- | --- | --- | --- |
| Expressed | Emotion | Fear | Happy | Neutral | Sad | Surprise |
|  | Fear |  | 1.19% | 1.27% | 1.50% | 31.78% |
|  | Happy | .67% |  | 9.54% | 1.62% | 1.39% |
|  | Neutral | 1.42% | .74% |  | 6.39% | .93% |
|  | Sad | 7.16% | 1.78% | 10.94% |  | 1.45% |
|  | Surprise | 12.60% | 2.22% | 4.67% | .75% |  |

**Supplementary Table 6.** Confusion matrix of expressed and perceived unmasked facial expressions of emotions in males only (N=120) representing the percentage of misattribution errors.

| Error % | Perceived | | | | | |
| --- | --- | --- | --- | --- | --- | --- |
| Expressed | Emotion | Fear | Happy | Neutral | Sad | Surprise |
|  | Fear |  | .84% | 3.64% | 11.11% | 21.96% |
|  | Happy | .80% |  | 1.57% | 1.93% | 1.71% |
|  | Neutral | 1.27% | .66% |  | 5.68% | 1.93% |
|  | Sad | 15.84% | 1.53% | 5.17% |  | 2.33% |
|  | Surprise | 16.93% | 2.37% | 1.46% | 1.27% |  |

**Supplementary Table 7.** Confusion matrix of expressed and perceived masked facial expressions of emotions in males only (N=120) representing the percentage of misattribution errors.

| Error % | Perceived | | | | | |
| --- | --- | --- | --- | --- | --- | --- |
| Expressed | Emotion | Fear | Happy | Neutral | Sad | Surprise |
|  | Fear |  | 1.35% | 1.75% | 1.50% | 28.21% |
|  | Happy | .47% |  | 10.66% | 1.80% | 2.28% |
|  | Neutral | 1.88% | .89% |  | 4.84% | .99% |
|  | Sad | 6.51% | 2.01% | 14.17% |  | 1.94% |
|  | Surprise | 11.24% | 2.05% | 4.69% | .76% |  |

**Supplementary Table 8.** Regression analyses for unmasked stimuli.

|  | **B** | **SE B** | ***ß*** | ***t*** | ***p*** | ***R^2^*** | ***N*** |
| --- | --- | --- | --- | --- | --- | --- | --- |
| **Total RT** | | |  |  |  |  |  |
| Overall Model | |  |  |  | < .001*** | .142 | 469 |
| Age | 76.029 | 9.560 | .363 | 7.952 | < .001*** |  |  |
| Sex | -18.286 | 9.184 | -.087 | -1.991 | .047* |  |  |
| ULS | -24.904 | 11.023 | -.119 | -2.259 | .024* |  |  |
| PSS | 17.638 | 14.120 | .084 | 1.249 | .212 |  |  |
| PHQ-9 | -3.907 | 11.172 | -.019 | -.350 | .727 |  |  |
| GAD-7 | 8.223 | 14.325 | .039 | .574 | .566 |  |  |
| **Fear RT** | | | | | |  |  |
| Overall Model | |  |  |  | < .001*** | .048 | 468 |
| Age | .031 | .004 | .324 | 7.004 | < .001*** |  |  |
| Sex | -.011 | .004 | -.119 | -2.680 | .008** |  |  |
| ULS | -.009 | .005 | -.093 | -1.733 | .084 |  |  |
| PSS | .006 | .007 | .062 | .906 | .366 |  |  |
| PHQ-9 | -.003 | .005 | -.032 | -.590 | .556 |  |  |
| GAD-7 | .006 | .007 | .067 | .966 | .334 |  |  |
| **Happy RT**  Logarithmic transformation | | | | | | |  |
| Overall Model | |  |  |  | <.001*** | .118 | 469 |
| Age | .030 | .004 | .314 | 6.730 | <.001*** |  |  |
| Sex | -.011 | .004 | -.113 | -2.519 | .012** |  |  |
| ULS | -.010 | .005 | -.106 | -1.969 | .05* |  |  |
| PSS | .006 | .007 | .065 | .945 | .345 |  |  |
| PHQ-9 | -.003 | .005 | -.026 | -.487 | .627 |  |  |
| GAD-7 | .006 | .007 | .065 | .933 | .351 |  |  |
| **Neutral RT**  Logarithmic transformation | | | | |  |  |  |
| Overall Model | |  |  |  | <.001*** | .089 | 469 |
| Age | .025 | .004 | .299 | 6.347 | <.001*** |  |  |
| Sex | -.007 | .004 | -.085 | -1.871 | .062 |  |  |
| ULS | -.006 | .005 | -.071 | -1.305 | .193 |  |  |
| PSS | .008 | .006 | .097 | 1.389 | .165 |  |  |
| PHQ-9 | -.001 | .005 | -.007 | -.133 | .894 |  |  |
| GAD-7 | .004 | .006 | .045 | .642 | .521 |  |  |
| **Sad RT**  Logarithmic transformation | | | |  |  |  |  |
| Overall Model | |  |  |  | <.001*** | .055 | 469 |
| Age | .017 | .004 | .214 | 4.455 | <.001*** |  |  |
| Sex | -.004 | .004 | -.056 | -1.219 | .223 |  |  |
| ULS | -.009 | .004 | -.115 | -2.077 | .038* |  |  |
| PSS | .005 | .006 | .067 | .950 | .343 |  |  |
| PHQ-9 | .002 | .004 | .020 | .365 | .715 |  |  |
| GAD-7 | .001 | .006 | .008 | .109 | .914 |  |  |
| **Surprise RT** | | | | |  |  |  |
| Overall Model | |  |  |  | <.001*** | .123 | 467 |
| Age | 87.803 | 12.535 | .324 | 7.004 | <.001*** |  |  |
| Sex | -22.037 | 12.058 | -.081 | -1.828 | .068 |  |  |
| ULS | -27.301 | 14.444 | -.101 | -1.890 | .059 |  |  |
| PSS | 2.144 | 18.504 | .074 | 1.089 | .316 |  |  |
| PHQ-9 | -1.829 | 14.619 | -.007 | -.125 | .821 |  |  |
| GAD-7 | -6.787 | 18.770 | -.025 | -.362 | .887 |  |  |

*Note:* ***p <.001 ; **p<.01 ; *<.05

|  | **B** | **SE B** | ***ß*** | ***t*** | ***p*** | ***R^2^*** | ***N*** |  |
| --- | --- | --- | --- | --- | --- | --- | --- | --- |
| **Total Accuracy**  Normality violated  (results before transformation are reported) | | | |  |  |  |  | |
| Overall Model | |  |  |  | <.001*** | .049 | 469 | |
| Age | -.796 | .496 | -.077 | -1.606 | .109 |  |  |  |
| Sex | 2.211 | .477 | .214 | 4.641 | <.001*** |  |  |  |
| ULS | .109 | .572 | .011 | .190 | .850 |  |  |  |
| PSS | -.369 | .733 | -.036 | -.504 | .614 |  |  |  |
| PHQ-9 | -.196 | .580 | -.019 | -.337 | .736 |  |  |  |
| GAD-7 | -.132 | .743 | -.013 | -.178 | .859 |  |  |  |
| **Fear Accuracy**  Logarithmic transformation | | | | | |  |  | |
| Overall Model | |  |  |  | <.001*** | .047 | 469 | |
| Age | -.021 | .008 | -.118 | -2.453 | .015* |  |  |  |
| Sex | .032 | .008 | .182 | 3.926 | <.001*** |  |  |  |
| ULS | -.002 | .010 | -.011 | -.204 | .839 |  |  |  |
| PSS | -.022 | .012 | -.123 | -1.730 | .084 |  |  |  |
| PHQ-9 | .003 | .010 | .018 | .312 | .755 |  |  |  |
| GAD-7 | -.004 | .013 | -.020 | -.282 | .778 |  |  |  |
| **Neutral Accuracy**  Normality violated  (results before transformation are reported) | | | |  |  |  |  |  |
| Overall Model | |  |  |  | .003** | .041 | 469 |  |
| Age | -.482 | .133 | -.174 | -3.612 | <.001*** |  |  |  |
| Sex | .206 | .128 | .075 | 1.609 | .108 |  |  |  |
| ULS | -.030 | .154 | -.011 | -.198 | .843 |  |  |  |
| PSS | -.192 | .197 | -.070 | -.975 | .330 |  |  |  |
| PHQ-9 | -.035 | .156 | -.013 | -.226 | .821 |  |  |  |
| GAD-7 | .280 | .200 | .101 | 1.399 | .162 |  |  |  |
| **Sad Accuracy**  Normality violated  (results before transformation are reported) | | | | | | |  | |
| Overall Model | |  |  |  | <.001*** | .049 | 469 | |
| Age | .539 | .197 | .132 | 2.735 | .006** |  |  |  |
| Sex | .618 | .189 | .151 | 3.263 | .001** |  |  |  |
| ULS | .156 | .227 | .038 | .685 | .494 |  |  |  |
| PSS | -.317 | .291 | -.077 | -1.087 | .278 |  |  |  |
| PHQ-9 | -.304 | .230 | -.074 | -1.319 | .188 |  |  |  |
| GAD-7 | .164 | .295 | .040 | .555 | .579 |  |  |  |
| **Surprise Accuracy**  Logarithmic transformation | | | | | |  |  | |
| Overall Model | |  |  |  | .001** | .045 | 469 | |
| Age | -.008 | .005 | -.073 | -1.517 | .13 |  |  |  |
| Sex | .016 | .005 | .148 | 3.189 | .002** |  |  |  |
| ULS | .002 | .006 | .017 | .298 | .766 |  |  |  |
| PSS | .016 | .008 | .141 | 1.984 | .048 |  |  |  |
| PHQ-9 | .001 | .006 | .012 | .207 | .836 |  |  |  |
| GAD-7 | -.012 | .008 | -.110 | -1.530 | .127 |  |  |  |

*Note:* **p <.001. *<.05

|  | **B** | **SE B** | ***ß*** | ***t*** | ***p*** | ***R^2^*** | ***N*** |
| --- | --- | --- | --- | --- | --- | --- | --- |
| **Total Performance**  Normality violated  (results before transformation are reported) | | | | | | |  |
| Overall Model | |  |  |  | < .001*** | .102 | 469 |
| Age | -.440 | .076 | -.271 | -5.790 | < .001*** |  |  |
| Sex | .302 | .073 | .185 | 4.129 | < .001*** |  |  |
| ULS | .130 | .088 | .080 | 1.476 | .140 |  |  |
| PSS | -.120 | .112 | -.074 | -1.069 | .286 |  |  |
| PHQ-9 | .000 | .089 | .000 | -.003 | .997 |  |  |
| GAD-7 | -.052 | .114 | -.032 | -.457 | .648 |  |  |
| **Fear Performance**  Normality violated  (results before transformation are reported) | | | | | | |  |
| Overall Model | |  |  |  | .004** | .040 | 468 |
| Age | -.306 | .080 | -.186 | -3.837 | <.001*** |  |  |
| Sex | .146 | .077 | .088 | 1.900 | .058 |  |  |
| ULS | .042 | .092 | .026 | .460 | .646 |  |  |
| PSS | -.136 | .118 | -.082 | -1.153 | .250 |  |  |
| PHQ-9 | .091 | .093 | .055 | .978 | .329 |  |  |
| GAD-7 | -.058 | .119 | -.035 | -.486 | .627 |  |  |
| **Happy Performance**  Normality violated  (results before transformation are reported) | | | | | | |  |
| Overall Model | |  |  |  | <.001*** | .094 | 469 |
| Age | -.450 | .078 | -.270 | -5.752 | <.001*** |  |  |
| Sex | .262 | .075 | .157 | 3.480 | <.001*** |  |  |
| ULS | .148 | .090 | .089 | 1.641 | .101 |  |  |
| PSS | -.094 | .116 | -.057 | -.815 | .416 |  |  |
| PHQ-9 | .037 | .091 | .022 | .405 | .686 |  |  |
| GAD-7 | -.159 | .117 | -.095 | -1.357 | .175 |  |  |
| **Neutral Performance**  Normality violated  (results before transformation are reported) | | | | | | |  |
| Overall Model | |  |  |  | <.001*** | .089 | 469 |
| Age | -.490 | .079 | -.292 | -6.201 | <.001*** |  |  |
| Sex | .159 | .076 | .094 | 2.090 | .037* |  |  |
| ULS | .057 | .091 | .034 | .621 | .535 |  |  |
| PSS | -.159 | .117 | -.095 | -1.361 | .174 |  |  |
| PHQ-9 | -.007 | .092 | -.004 | -.078 | .938 |  |  |
| GAD-7 | .061 | .118 | .036 | .517 | .605 |  |  |
| **Surprise Performance**  Normality violated  (results before transformation are reported) | | | | | |  |  |
| Overall Model |  |  |  |  | <.001*** | .092 | 467 |
| Age | -.415 | .079 | -.248 | -5.258 | <.001*** |  |  |
| Sex | .215 | .076 | .128 | 2.834 | .005** |  |  |
| ULS | .097 | .091 | .058 | 1.068 | .286 |  |  |
| PSS | .075 | .117 | .045 | .645 | .519 |  |  |
| PHQ-9 | .010 | .092 | .006 | .110 | .912 |  |  |
| GAD-7 | -.081 | .118 | -.049 | -.687 | .492 |  |  |

*Note:* ***p <.001 ; **p<.01 ; *<.05

**Supplementary Table 9.** Regression analyses for masked stimuli.

|  | **B** | **SE B** | ***ß*** | ***t*** | ***p*** | ***R^2^*** | ***N*** |
| --- | --- | --- | --- | --- | --- | --- | --- |
| **Total RT** | | | | | | |  |
| Overall Model | |  |  |  | <.001*** | .147 | 469 |
| Age | 82.454 | 9.838 | .382 | 8.381 | <.001*** |  |  |
| Sex | -18.794 | 9.451 | -.087 | -1.989 | .047* |  |  |
| ULS | 6.541 | 11.344 | .030 | .577 | .564 |  |  |
| PSS | 1.967 | 14.530 | .009 | .135 | .892 |  |  |
| PHQ-9 | -1.549 | 11.497 | -.049 | -.918 | .359 |  |  |
| GAD-7 | 9.813 | 14.741 | .045 | .666 | .506 |  |  |
| **Fear RT**  Logarithmic transformation | | | | |  |  |  |
| Overall Model | |  |  |  | <.001*** | .053 | 454 |
| Age | .020 | .004 | .224 | 4.574 | <.001*** |  |  |
| Sex | -.005 | .004 | -.054 | -1.138 | .256 |  |  |
| ULS | .000 | .005 | -.005 | -.097 | .923 |  |  |
| PSS | .000 | .006 | .002 | .021 | .983 |  |  |
| PHQ-9 | -.001 | .005 | -.011 | -.198 | .843 |  |  |
| GAD-7 | .001 | .007 | .011 | .153 | .879 |  |  |
| **Happy RT**  Logarithmic transformation | | | | | |  |  |
| Overall Model | |  |  |  | <.001*** | .119 | 468 |
| Age | .031 | .004 | .333 | 7.191 | <.001*** |  |  |
| Sex | -.011 | .004 | -.124 | -2.779 | .006** |  |  |
| ULS | 2.651E-05 | .005 | .000 | .005 | .996 |  |  |
| PSS | -.002 | .006 | -.026 | -.378 | .706 |  |  |
| PHQ-9 | .000 | .005 | .005 | .096 | .924 |  |  |
| GAD-7 | .009 | .006 | .092 | 1.326 | .186 |  |  |
| **Neutral RT**  Logarithmic transformation | | | | | |  |  |
| Overall Model | |  |  |  | <.001*** | .095 | 469 |
| Age | .027 | .004 | .314 | 6.700 | <.001*** |  |  |
| Sex | -.003 | .004 | -.035 | -.773 | .440 |  |  |
| ULS | .004 | .005 | .049 | .905 | .366 |  |  |
| PSS | .004 | .006 | .046 | .663 | .508 |  |  |
| PHQ-9 | -.004 | .005 | -.048 | -.879 | .380 |  |  |
| GAD-7 | .000 | .006 | .002 | .032 | .974 |  |  |
| **Sad RT**  Logarithmic transformation | | | | | |  |  |
| Overall Model | |  |  |  | <.001*** | .098 | 465 |
| Age | .024 | .004 | .286 | 6.079 | <.001*** |  |  |
| Sex | -.012 | .004 | -.150 | -3.313 | .001** |  |  |
| ULS | -.005 | .004 | -.055 | -1.022 | .307 |  |  |
| PSS | .006 | .006 | .068 | .976 | .329 |  |  |
| PHQ-9 | -.003 | .005 | -.039 | -.707 | .480 |  |  |
| GAD-7 | .005 | .006 | .055 | .779 | .437 |  |  |
| **Surprise RT** | | | | | | |  |
| Overall Model | |  |  |  | <.001*** | .125 | 465 |
| Age | 87.606 | 13.131 | .309 | 6.672 | <.001*** |  |  |
| Sex | -23.824 | 12.669 | -.084 | -1.880 | .061 |  |  |
| ULS | 24.626 | 15.172 | .087 | 1.623 | .105 |  |  |
| PSS | -21.682 | 19.424 | -.077 | -1.116 | .265 |  |  |
| PHQ-9 | -22.698 | 15.335 | -.080 | -1.480 | .140 |  |  |
| GAD-7 | 7.121 | 19.682 | .025 | .362 | .718 |  |  |

*Note:* ***p <.001 ; **p<.01. *<.05

|  | **B** | **SE B** | ***ß*** | ***t*** | ***p*** | ***R^2^*** | ***N*** |
| --- | --- | --- | --- | --- | --- | --- | --- |
| **Total Accuracy**  Logarithmic transformation | | | | | | |  |
| Overall Model | |  |  |  | .002** | .045 | 469 |
| Age | -.015 | .004 | -.162 | -3.366 | .001** |  |  |
| Sex | .013 | .004 | .144 | 3.108 | .002** |  |  |
| ULS | -.001 | .005 | -.013 | -.228 | .820 |  |  |
| PSS | -.001 | .006 | -.009 | -.131 | .896 |  |  |
| PHQ-9 | -.005 | .005 | -.051 | -.902 | .368 |  |  |
| GAD-7 | -.002 | .006 | -.024 | -.327 | .744 |  |  |
| **Fear Accuracy**  Normality violated  (results before transformation are reported) | | | | |  |  |  |
| Overall Model | |  |  |  | .014* | .034 | 469 |
| Age | -.672 | .252 | -.129 | -2.663 | .008** |  |  |
| Sex | .252 | .242 | .048 | 1.040 | .299 |  |  |
| ULS | .146 | .291 | .028 | .502 | .616 |  |  |
| PSS | -.810 | .372 | -.156 | -2.175 | .03* |  |  |
| PHQ-9 | -.538 | .295 | -.103 | -1.826 | .068 |  |  |
| GAD-7 | .254 | .378 | .049 | .673 | .502 |  |  |
| **Sad Accuracy**  Logarithmic transformation | | | | |  |  |  |
| Overall Model | |  |  |  | .001*** | .049 | 465 |
| Age | -.008 | .009 | -.042 | -.863 | .388 |  |  |
| Sex | .036 | .009 | .183 | 3.941 | < .001*** |  |  |
| ULS | .020 | .011 | .102 | 1.826 | .068 |  |  |
| PSS | .009 | .014 | .046 | .651 | .515 |  |  |
| PHQ-9 | -.010 | .011 | -.053 | -.942 | .346 |  |  |
| GAD-7 | -.014 | .014 | -.071 | -.978 | .329 |  |  |

*Note:* ***p <.001 ; **p<.01 ; *<.05

|  | **B** | **SE B** | ***ß*** | ***t*** | ***p*** | ***R^2^*** | ***N*** |
| --- | --- | --- | --- | --- | --- | --- | --- |
| **Fear Performance**  Normality violated  (results before transformation are reported) | | | |  |  |  |  |
| Overall Model | |  |  |  | .006** | .040 | 454 |
| Age | -.326 | .078 | -.206 | -4.180 | <.001*** |  |  |
| Sex | .061 | .076 | .038 | .805 | .421 |  |  |
| ULS | .008 | .090 | .005 | .092 | .927 |  |  |
| PSS | -.147 | .115 | -.093 | -1.284 | .200 |  |  |
| PHQ-9 | -.036 | .092 | -.022 | -.392 | .696 |  |  |
| GAD-7 | .057 | .116 | .036 | .489 | .625 |  |  |
| **Neutral Performance**  Normality violated  (results before transformation are reported) | | | | | | |  |
| Overall Model | |  |  |  | <.001*** | .069 | 469 |
| Age | -.452 | .080 | -.267 | -5.615 | <.001*** |  |  |
| Sex | .068 | .077 | .040 | .883 | .378 |  |  |
| ULS | -.069 | .093 | -.041 | -.740 | .460 |  |  |
| PSS | -.133 | .119 | -.079 | -1.121 | .263 |  |  |
| PHQ-9 | .095 | .094 | .056 | 1.008 | .314 |  |  |
| GAD-7 | -.005 | .121 | -.003 | -.039 | .969 |  |  |
| **Sad Performance**  Normality violated  (results before transformation are reported) | | | | | | |  |
| Overall Model | |  |  |  | <.001*** | .101 | 465 |
| Age | -.351 | .075 | -.219 | -4.672 | <.001*** |  |  |
| Sex | .366 | .072 | .228 | 5.044 | <.001*** |  |  |
| ULS | .155 | .087 | .097 | 1.784 | .075 |  |  |
| PSS | -.047 | .111 | -.030 | -.428 | .669 |  |  |
| PHQ-9 | -.024 | .088 | -.015 | -.267 | .790 |  |  |
| GAD-7 | -.122 | .113 | -.076 | -1.082 | .280 |  |  |

*Note:* ***p <.001 ; **p<.01 ; *<.05
